# Remote sensing image information extraction based on Compensated Fuzzy Neural Network and big data analytics

**DOI:** 10.1186/s12880-024-01266-9

**Published:** 2024-04-10

**Authors:** Rui Sun, Zhengyin Zhang, Yajun Liu, Xiaohang Niu, Jie Yuan

**Affiliations:** 1 Yellow River Conservancy Technical Institute, Kaifeng, Henan 475001 China; 2 China University of Mining and Technology, Xuzhou, China; 3 Guangdong Nuclear Industry Geology Bureau Surveying and Mapping Institute, Guangzhou, Guangdong 510800 China; 4 POWERCHINA HARBOUR Co.,LTD, Tianjin, 300450 China; 5 School of Information Engineering, Minzu University of China, Beijing, 100081 China

**Keywords:** Information retrieval, Feature information extraction, Remote sensing, Fuzzy logic, Medical imaging AI systems, Big data analytics

## Abstract

Medical imaging AI systems and big data analytics have attracted much attention from researchers of industry and academia. The application of medical imaging AI systems and big data analytics play an important role in the technology of content based remote sensing (CBRS) development. Environmental data, information, and analysis have been produced promptly using remote sensing (RS). The method for creating a useful digital map from an image data set is called image information extraction. Image information extraction depends on target recognition (shape and color). For low-level image attributes like texture, Classifier-based Retrieval(CR) techniques are ineffective since they categorize the input images and only return images from the determined classes of RS. The issues mentioned earlier cannot be handled by the existing expertise based on a keyword/metadata remote sensing data service model. To get over these restrictions, Fuzzy Class Membership-based Image Extraction (FCMIE), a technology developed for Content-Based Remote Sensing (CBRS), is suggested. The compensation fuzzy neural network (CFNN) is used to calculate the category label and fuzzy category membership of the query image. Use a basic and balanced weighted distance metric. Feature information extraction (FIE) enhances remote sensing image processing and autonomous information retrieval of visual content based on time-frequency meaning, such as color, texture and shape attributes of images. Hierarchical nested structure and cyclic similarity measure produce faster queries when searching. The experiment’s findings indicate that applying the proposed model can have favorable outcomes for assessment measures, including Ratio of Coverage, average means precision, recall, and efficiency retrieval that are attained more effectively than the existing CR model. In the areas of feature tracking, climate forecasting, background noise reduction, and simulating nonlinear functional behaviors, CFNN has a wide range of RS applications. The proposed method CFNN-FCMIE achieves a minimum range of 4–5% for all three feature vectors, sample mean and comparison precision-recall ratio, which gives better results than the existing classifier-based retrieval model. This work provides an important reference for medical imaging artificial intelligence system and big data analysis.

## Introduction

It is important to investigate medical imaging AI systems and big data analytics. With the rapid development of remote sensing technology, remote sensing images, as typical applications of medical imaging AI systems and big data analytics, play a huge role in environmental monitoring, economic investigation, natural disaster prediction, environmental degradation monitoring, etc. Several efforts have been made to develop an effective method for information extraction operations in RS. The availability of many high-resolution (HR) photos provides a benefit for more accurate information extraction by creating sophisticated categorization methods. The process of RS classification is complicated and takes various elements into account. First, more information on the relative usefulness of various forms is required to better assist the user in making judgments regarding image classification. Utilizing Content-Based Image Extraction (CBIE) in CBRS. CBIE is a method that searches a database of images using visual content. CBIE uses optical characteristics like color and texture to describe pictures. CBIE is a subset of imaging, and computer vision derives many approaches from those disciplines. In the first place, neural networks are data-driven self-adaptive methods that can change based on the data without requiring user-defined or distributional form specifications for the underlying model. When remote sensing data is provided in digital format, neural networks, a computer technology, must be used for digital processing and analysis. Fuzzy concepts and neural network technologies are interacting and combining due to development. There is a technical problem with the typical neural network. For instance, the ideas are unsure about how to choose a fuzzy membership function, fuzzy logic reasoning, and imprecise optimized calculation. Due to the neural network’s incredibly effective and precise classification capabilities, both recall rate and retrieval time have been markedly enhanced.

A Compensated Fuzzy Neural Network (CFNN) offered as a solution to this issue follows the integration of fuzzy theory with the neural network. It has compensating fuzzification capabilities and quick study calculation capabilities. CFNN is a hybrid system that combines neural networks and balanced fuzzy logic. It can strengthen the image stability of the process and increase the fault tolerance of the extraction process. Agriculture, temperature, water bodies, and many more fields use remote sensing as a key means of earth monitoring without direct physical contact [1]. Due to the difficulty in managing data queries due to the sheer volume of data from several sources, current data organization, storage, and management solutions cannot satisfy application requirements [2]. Massive RS photos are essential for many applications, particularly combustion RS images, as they capture the planet’s surface enhancement, spatial guidance, disaster relief, and more. Early on, RS information is one type of pricey and in-demand resource [3]. The job of RS-Image Retrieval (RS-IR), which seeks to find a group of objects that are comparable to a given query image, is crucial in remote sensing applications [4]. One of the fundamental problems is usually the segmentation of remote-sensing images with imbalanced samples [5]. The handling of elevated RS for high-resolution images has become difficult due to the rapid development of their volume and resolution.

The customary content-based Remote-Sensing Image Extraction (CBRSIE) technologies [6]. A significant result is Content-Based Image Extraction (CBIE), which tries to analyze photographs using features of an image comparable to the query image the user submitted. With this method, an image’s description comprises dynamically retrieved visual elements, including hue, structure, and shape [7]. It is only necessary to normalize one channel when using a grayscale image. However, if normalizing a RedGreenBlue(RGB 3 channels), we should use the same standards for each channel [8]. Unorganized image entity information and structured metadata comprise the majority of remote sensing data. Due to the peculiarities of huge geographic data realization, the ability to aggregate all entity data is governed by several factors, including the access mechanism, available storage space, labor costs, etc. [9]. Structure extraction from remote sensing pictures can be done in several ways, including extracting features based on prior knowledge of things like strong edges, shape designs, roof colors, shadows, etc. [10]. The CCFN can carry out compensated fuzzy reasoning and features a fleet self-study algorithm [11]. With the number of images expanding rapidly, Content-Based Image Extraction (CBIE) has garnered more attention [12]. Depending on the IF-THEN implication, a fuzzy logic system concludes. Based on the study’s findings, control rules are developed concurrently, and class labels are constructed. The produced model’s functionality is evaluated for accuracy [13].

The planned work’s contribution is as follows:

- A unique model is recommended for image information extraction from an RS field using the CBIE technique called FMCIE to handle low-level images like texture from the RS sector.
- Integrated proposed FMCIE with CFNN model is used to compute the query image’s class label and fuzzy class membership.
- Feature Information Extraction and Hierarchically nested structures are analyzed to get similar recurrent images to form a query database. The outcomes reduce the retrieval speed of pictures from the RS field.
- It shows the ratio of coverage, average mean precision, and recall. Comparison of the proposed work is better than other existing Classifier based Retrieval methods.

The planned work is structured as follows: Categorization of earlier knowledge describing the relationship between RS and CFNN for efficient Retrieval of images and its similarities using various methods are discussed in Sect. 2. To extract similar images from the stored dataset of the RS field, Sect. 3 introduces the CFNN and suggests an FCMIE model for the system. The experimental findings for CBIR using FMCIE models are presented in Sect. 4, which also provides the best precision, recall, and coverage ratio. Compared to traditional Classifier-based models, average Mean Precision (AMP) from RS pictures. The conclusion and the potential for future improvement in the RS image analysis using Compensated Fuzzy Neural Networks are discussed in Sect. 5.

## Related work

Shao et al. (2019) suggested a method to simultaneously distinguish light clouds and quasi-components in RS photos using a Multiresolution Functionalities Convolutional Neural Network (MF-CNN) [14]. In terms of proper identification rate, the self-contrast approach performs the worst, and the majority of values across the board for cloud detection are less than 0.75, rates more favorable in terms of the good detection rate.

Tang et al. (2018) suggested the Features Extraction method and which is created using the Bag-Of-Words (BOW) paradigm and deep learning technology [15]. The learning method is divided into two steps: building features and learning picture descriptors. Over 50 countries are represented in the photographs, all taken from Google Earth. The original range of each image is 256 for 256, and the pixel ranges from 29 to 0.3 m.

Ghrabat et al. (2019) elaborated an innovative SVM-based Convolutional Neural Network(SVMB-CNN) is utilized to classify the characteristics after they have been optimized using a modified evolutionary approach [16]. The undesirable data in the dataset are removed using a Gaussian filtering approach. The experimental outcomes of the Corel 1K dataset’s precision, recall, and retrieval rate, which has respective values of 99, 97, and 95% better results, are obtained from our proposed model.

Desai et al. (2021) proposed a Convolution for Neural-Networks (CNN) based Support Vector Machines (SVM) are utilized to provide an effective deep learning framework for quick picture retrieval [17]. The suggested architecture uses SVM for classification and CNN to extract features. The outcome of this proposed work is: For the confusion matrix, the category with the highest accuracy rate (95%) and genuine positive sign (45%) is Animals, whereas the class with the lowest accuracy rate (60%) and real positive value (30) is Hills.

Sezavar et al. (2019) defined a novel search technique called the Modified Grasshopper Optimization Algorithm (MGOA) suggested to solve the modeled problem and effectively locate related photos [18]. It is noted that the proposed strategy achieves the best P(0.5) and P(1) scores at 93 and 81%, respectively. The proposed approach yields the best results, whose vector size is above 1024, whereas the proposed approach that performs better as a feature vector that is 70 bytes in size.

Ghrabat et al. (2019) proposed the Multiple Ant Colony Optimization (MACO) method to locate pertinent characteristics, and it is used with all of the features [19]. The relevant factors are used for the Greedy learning of the Deep Boltzmann Machine classifier (GD-BM). Compared to current methods like the a priori classification algorithm, the GD-BM offers a 28% improvement in accuracy. The system now only takes about 3.75 ms for each image, which is better than the current system’s time usage.

Guanglong et al. (2020) presented an online cognitive angular position error compensation method that utilizes incremental learning to decrease joint angle error [20]. This method predicts and updates the compensation in real-time using the Self-Feedback Incremental- FNN (SFIFN). The efficiency of the suggested method for correcting joint angle inaccuracy is demonstrated. The proposed model decreased the inaccuracy by around 0.02 degrees compared to the direct compensation method.

Zhang, Jin, et al. (2021) proposed a compensation fuzzy neural network technique that incorporates the fuzzy compensation algorithm and recurrent neural network for detecting and recognizing table tennis’s technical and strategic indicators [5]. The information and prediction outcomes show that the model’s accuracy rises with increasing the number of input coordinates. The acceptable error range in a practical application is less than 40 mm when the input data length is 30 mm.

A novel loss function-based picture segmentation technique built on ConvNet was created and tested by Chen et al. (2021) [21] to provide a more accurate quantitative evaluation of bone metastases because it can measure activity in overlapped structures. The suggested quasi-Convolution model, trained using models formulated, produced Coefficients of Dice Similarity (CDS) of 0:70 and 0:80 for tumor and bones segmentation in Quantitative analysis of Bone based Single-Photon Emission in Computed Tomography QBSPECT/CT.

Chen et al. (2021) suggested a method based on fuzzy neural networks that examine the factors contributing to music instruction’s eligibility in universities and colleges [22]. The rate of low teaching quality indicated by 30 polytechnic institutions and 30 universities using the fuzzy control method for incident detection ratio in music training effectiveness of 81.7% of colleges and universities are comprehensive.

Ai et al. (2019) elaborated a technique for the automatic control of the antimony flotation process, and an adaptive fuzzy neural network control strategy based on data was created [23]. The LSTM (Long ShortTerm Memory) network and the Radial Base Function Neural Network (RBFNN)are integrated into the data-driven model. The outcome of our strategy reduced the standard deviation by 0.1809 and 0.2589 sample images compared to the other two control methods.

Ye et al. (2018) proposed CNN fundamental features and a weighted range-based retrieval approach [24]. Before extracting the pre-trained network is initially perfectly alright to use some scanned copy from the objective data set before CNN features and labels the images in the retrieved collected data. When there are more than ten training images, our methods produce better outcomes than the alternatives. With the suggested methods, the standard deviation of the search sequence number is 0.04 lowest.

Summary of this session: The work included image information extraction in the remote sensing field, and integration with the fuzzy neural network is assessed and compiled. This literature survey refers to efficient and improved methods Content-Based Image Retrieval (CBIR) techniques and QBSPECT/CT, LSTM, CNN, Fuzzy logic, and Classifier based Retrieval algorithms for mitigating efficient image retrieval by various researchers and authors. Also, did a literature survey to find published models for image handling and management in addition to feature extraction strategies and evaluation metrics. Comparison of platforms like CR, SFIFN, GA, MGOA, and another BOW-based CNN, including effective image information extraction.

## Proposed work

From medical imaging AI systems, the major challenge with a Content-Based Image Extraction system (CBIE) is to retrieve the image information features from the RS that accurately represent the contents of images in a database. A thorough analysis of the image feature information performance is necessary for this extraction. Colour and texture feature extraction is part of CBIE. The color histogram, color correlogram, and color moment are frequently used color properties that are compared. Image information extraction effectiveness increases when texture and color features are integrated. Also mentioned are the similarity measurement criteria used to find matches and retrieve the correctness of the images in an RS field. Feature Information Extraction describes utilizing a computer to collect visual data and determine if each object’s pixels are included in an extracting feature. The edge characteristic, analysis of the corner feature’s present location, color highlight, texture showcase, and edge function are some of the picture features employed in several feature extraction methods in this study. A unique model is recommended for image information extraction from an RS field using the CBIR technique called FMCIE to handle low-level images like texture from the RS sector. Integrated proposed FMCIE with CFNN model is used to compute the query image’s class label and fuzzy class membership. Feature Information Extraction and Hierarchically nested structures are analyzed to get similar recurrent photos to form a query database. The outcomes reduce the retrieval speed of images from the RS field. It shows the ratio of coverage, Average Mean Precision, and recall Comparison of the proposed work is better than other existing Classifier-based retrieval methods.

**Fig. 1:**
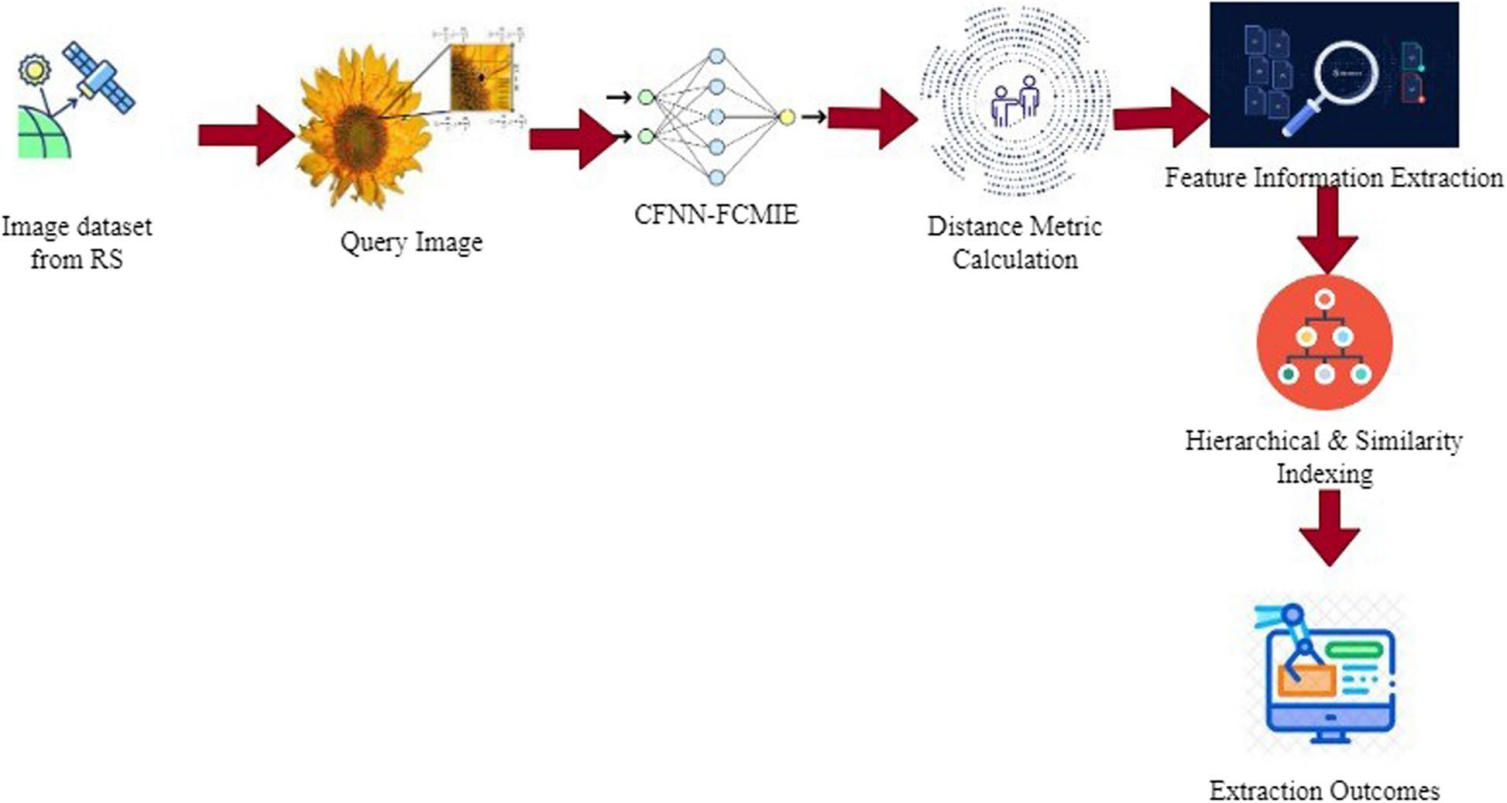
CFNN - FCMIE method for image information extraction from RS with medical imaging AI systems and big data analytics

In Fig. 1, the suggested model for the CBRS technique of image information extraction is depicted clearly with the CFNN-FMCIE method of information extraction process from RS. Initially, the freely available dataset is downloaded, and the image is taken from a stored database of RS. Then the query image is considered as input for the proposed model. The algorithm steps and formulas are explained clearly in that module. After that measure, the distance metric of the image is calculated with the necessary equation. FIE is used to extract information on image attributes like color, shape, and texture; the region is analyzed. The output is forwarded for hierarchical arrangement structures, and similarity measurement is taken for those images stored in a database. The experimental outcomes show the information attributes extracted from them and the retrieval ratio of coverage, Average Mean Precision, and recall of the proposed method are better than conventional models.

### CFNN for fuzzy class membership-based image extraction and big data analytics

The proposed FCMIE method can extract the image information using the convolution method. The data is a high-dimensional matrix with the corresponding width and length for the 3D image. It consists of two steps: Retrieval based on the class label and fuzzy class membership-based image extraction (FCMIE) of the query picture value. Secondly, weighted distance is measured when the search space is restricted to the classes. Multiple labeled image datasets and the related feature set are used to train a multi-layered fuzzy class neural network with one hidden layer. Statistical texture features are extracted using the wavelet transform. These feature sets are utilized to calculate the query image’s class membership value and the feature space distance between the query and database images. Maximum nonlinear separations between various made in the feature space are the objective of learning. The neural network requires 𝑑=20 cells at the input nodes (one cluster at the input layer is utilized as a biassed input) and c (number of output variable) nodes at the hidden layers to categorize feature maps of dimensions ten into the numeric count of classes. The buried layer of the network makes use of “$2d+1$” neurons. A 4-fold cross-validation approach is used to evaluate the image information extraction and retrieval speed performance.

FCMIE value of the image of $X$ having feature vector $\overrightarrow{f}\left(X\right)$ For the particular class, j is calculated by the equation 

1 $${\mu }_{j}\left(\overrightarrow{f}\left(X\right)\right)=\frac{{0}_{j}\left(\overrightarrow{f}\left(X\right)\right)}{\sum _{i=1}^{c}{0}_{i}\left(\overrightarrow{f}\left(X\right)\right)}$$

Where ${0}_{j}\left(\overrightarrow{f}\left(X\right)\right)$ represents the output layer called ${j}^{th}$ neuron for the input value $\overrightarrow{f}\left(X\right)$. ${\mu }_{j}\left(\overrightarrow{f}\left(X\right)\right)$ Represents the fuzzy class membership of image $X$ to the particular class outcome $j$. The outcome of FCMIE must satisfy the following constraints. Mean is represented by ${\mu }_{j}$. The output variable is represented by $c.$

2 $$0\le\,{\mu\,}_{j}\left(\overrightarrow{{f}_{1}}\left(X\right)\right)\le\,1\,\forall\,j\,ranges\,from\left[\,1\,to\,c\right]$$

3 $${\textstyle\sum_{j\geq1}^c}\mu_j\left(\overrightarrow{f_1}\left(X\right)\right)=1$$

**Fig. 2:**
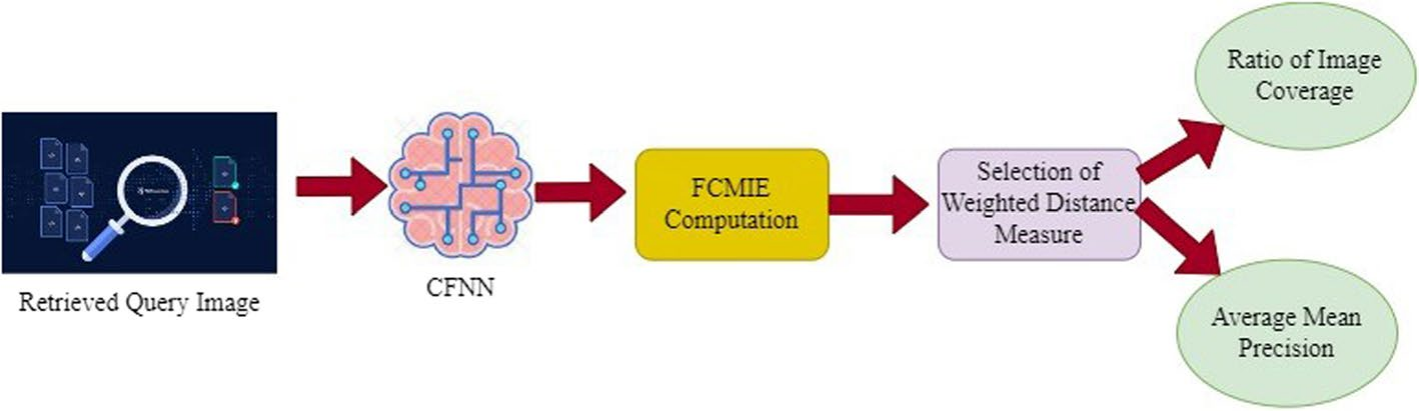
Class Membership-based FCMIE method image information extraction

The suggested method FCMIE in Fig. 2 uses a trained neural network to automatically determine the fuzzy Membership of the query image from the given feature set. The feature set mapping is not necessary for this. The suggested method minimizes the number of categories for learning by not requiring manual regrouping of perceptually related textures from distinct classes.

FCMIE Pseudocode for Image Information Extraction from RS.

1. Resize all images in each database, feed them to CFNN, and start training.
2. Save essential information features of images
3. Initialize FCMIE
4. If $${o}_{j}\left(\overrightarrow{f}\left(X\right)\right)\le\,t$$ 
5. Extract query features from a stored database
6. Distribute the images information randomly
7. Use class Measure for Weighted Distance else
8. Use Distance metric simple calculation, $d\left(X,{Y}_{j}\right)$ Endif.

Evaluating metrics performance is one of the most important steps in content-based RS image extraction. A wide range of system performance measurement techniques is applied. The performance techniques, recall, and Average Mean Precision was employed.

## Measure for weighted distance

The FCMIE, as mentioned earlier, method of image extraction then needs to take weighted distance measures when the space is restricted to the classes proposed by the Classifier; the classifier-based retrieval strategy provides 90% retrieval performance for each valid classification. However, the technique utterly fails to function in the event of misclassification. A weighted distance metric is suggested to consider retrieving efficiency in real and misclassification scenarios. For each proper classification Classifier, the goal is to apply a minimal penalty to all images within the same class and a comparatively bigger penalty to all the other category images in the database. When retrieving database images, the same sentence is applied to all of the images for each misclassification. Then the proposed weighted distance between two images in the feature vectors from the RS field is calculated by the equation

4 $${d}_{w}\left(X,{Y}_{j}\right)=\frac{1}{1+Z*{\mu }_{j}\left(\overrightarrow{f}\left(X\right)\right)}*d(X,{Y}_{j})$$

Where $Z$ represents the constant number that should not be negative. ${\mu }_{j}\left(\overrightarrow{f}\left(X\right)\right)$ Represents the fuzzy class membership of image $X$ to the particular class outcome $j$. $d(X,{Y}_{j})$ represents the distance between the $X\,and\,{Y}_{j}$  

### Ratio of coverage

The above metrics and weighted distance measures for statistical feature image vectors are analyzed. The approaches most frequently used to evaluate the efficacy of retrieval models have been recalling, precision, and recall precision. In particular, the user cannot calculate the recall index until they have viewed every relevant image, which is only achievable through a thorough search. The rate of uptake that can be used for RS image information extraction was utilized as the performance parameter in this study because the user could assess the effectiveness of the information extraction search progress. According to the equation below, this ratio of coverage can be determined.

5 $$RC=\left\{\begin{array}{l}\frac{n_{R_i}}{10i}\left(10i\leq R\right)\,and\\\frac{n_{R_i}}R(10i>R)\end{array}\right\}$$

Where $R$ is the count of overall related pictures in the stored component, ${n}_{{R}_{i}}$Is the count of related pictures in the first $10i$ pictures. When the outcome of the first condition $(10i\le R )$, the ratio of coverage is equal to the precision; when the ranges of $(10i>R),$ the percentage of capacity is similar to the recall. In this equation, the value of $i$ is taken as {1, 2, 3, 4, 5, 10, 20}.

#### Average mean precision

After calculating the Ratio of Coverage, the performance measure needs to calculate another metric named Average Mean Precision. The single-value metrics of precision and re-call-precision are based on the entire collection of photos supplied by the information retrieval. It is advisable to monitor the presentation range of the getting images for algorithms that produce a rated image sequence. The more relevant photos are ranked higher in the average precision index. The precisions calculated for each appropriate illustration in the ranking sequence are averaged to get this index. The mean precision scores for each search make up the average precision for a group of inquiries. It is determined as

6 $$AMP=\frac1{I_{rk}}\;{\textstyle\sum_{rk=1}^{I_s}}\frac{\rho_s}{\rho_{rk}}$$

where $rk$ is the rank of an image, ${I}_{r}$ represents the count of related pictures obtained, ${I}_{s}$ Denotes the count of relevant source pictures obtained, ${\rho }_{rk}$ Is the priority part in the relevant pictures that are obtained, and ${\rho }_{s}$ Is the rank number in the real related pictures that are obtained?

### Features information extraction (FIE)

After identifying the ratio of coverage and Average Mean Precision of a retrieved image by the above-mentioned weighted distance measure, then need to calculate Features Information Extraction (FIE) is the process of extracting characteristics from an image, such as its color, texture, shape, and edges. A combination of features is necessary to obtain reliable retrieval results; a single factor does not provide accurate results. The pre-processing and feature extraction stages can be used to categorize the tasks carried out by CBIE. Noise is removed during the pre-processing step, and specific object properties important for understanding the image are enhanced. Segmentation of the image is also used to distinguish objects from the backdrop of the picture. Shape, color, texture, and other features are employed to describe the contents of the image during the feature extraction stage. Moments, scatter plots, and automated manner are a few approaches that can be used to achieve the color aspect. It is possible to implement the texture aspect via vector quantization or transforms. At this stage, highly similar image information extraction is also carried out. The following formulation can be used to measure similarity. The recall and precision of the system’s retrieval performance can be evaluated. Accuracy examines the system’s ability to recover only the relevant models, whereas recall evaluates the system’s capacity to retrieve all applicable models.

Distance metric based on weighted depth includes general structure of residual network, domain adaptation, inter-class adaptation of subdomain, intra-class adaptation of subdomain and filtering mechanism (DF). On the premise that the source domain and the target domain contain the same category, the residual network is used to learn and classify the invariant features of the samples in the source domain and the target domain. The loss is used to dynamically adjust the domain adaptation to increase the credibility of the pseudo-label and ensure that the samples are not too close to distinguish. On this basis, the intra-class and inter-class adaptation of subdomains are adopted to reduce the deviation of cross-domain conditional distribution, increase the inter-class distance of different categories and improve the classification accuracy.

Feature Information Extraction aims to divide the picture’s elements into various groups. Often, these subgroups consist of a single point, smooth curves, or a region. A variety of features typically describes the image. These features can be categorized using various criteria, including feature points, line features, and regional characteristics, depending on how they are represented in the image data. The depth recovery method relies on the scaling-composite scaling factor range of the picture to retrieve the desired data from the concept based on the attribute of the process and the information extraction of the targeted image in all domains. As shown by the following expression,

1. create Multiple blurred images using a synthesis weighting data model.

7 $$g\left(t\right)=\sqrt{s}f\left(\left[t-\tau\,\right]\right)$$

Where $\sqrt{s}$ Is the image time-frequency composite’s normalized factor a weighting distance metric.

2) Map just one function to the continuous evening image of the moment aggregate scaled 0 and then execute a period composites heavy transform 2D function $y\left(t\right)$ of the velocity and rhythm shift $a$ and $b$, as illustrated above.

3) changing the source picture’s attribute $f\left(t\right)$ By rephrasing the sentence to produce a variable’s time scale and time shift $a\,and\,b$.

8 $$a=\frac{1}{s}\,and\,b=\tau$$

4) Create a multi-frame period composite weighting signal form for the fuzzy image.

9 $$i\left(t\right)=\frac{1}{\sqrt{T}}rect\left(\frac{t}{T}\right)exp\left\{-j\left[2\pi k ln\left(1-\frac{t}{{t}_{0}}\right)\right]\right\}$$

With the condition of $rect\left(t\right)=1$ and $\left|t\right|\le \frac{1}{2}$

5) Signal of the period aggregate scaled inter fuzzy picture modulation law is a hyperbolic function.

10 $${f}_{i}\left(t\right)=\frac{K}{t-{t}_{0}} \text{a}\text{n}\text{d} \left|t\right|\le \frac{T}{2}$$

11 $$K=\frac{T{f}_{MAX}{f}_{MIN}}{B},{t}_{0}={f}_{0}T/B$$

Where ${f}_{0}$ represents the frequency of the central arithmetic value.

Therefore, the time-frequency composites weighting algorithm can better achieve the extraction technique of image features than the conventional time domain.

**Fig. 3:**
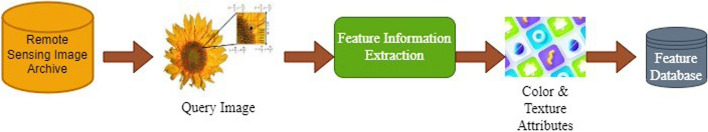
The framework of CFNN-based FIE of image retrieved from RS

From Fig. 3, the analysis of image information attributes stored in Low-level feature representation, which provides the basic details, is created by knowledge specialists and is frequently constructed by the channel colors or shape signals of data. RS photography consists of various features than original photos. The feature part is the basic building block despite being one of the most basic features, the spectral quality. It represents the reflectivity of the relevant regions for the environment factor by coding the important details.

#### FIE-Color

The color feature is the visual element that is most frequently employed in picture retrieval. The color feature is reasonably resistant to background issues. In a 3D image, every aspect can be used as a point. Color spaces that are frequently used are RGB. The R, G, denotes a color, and B is in the RGB color space, where R means the brightness of the red component, G indicates the strength of the green piece, and B is the sharpness of the blue part. The HSV color model characterizes colors according to their brightness and hues (Luminance). This model presents the link between colors in a more understandable manner. A color model describes a reference frame and a space inside it where every other color is represented by just one point. The below formula can be used to convert a pixel’s RGB representation into its HSV values:

12 $$S=1-\frac{3\left[min\left(RGB\right)\right]}{R+G+B}$$

13 $$V=\frac{R+G+B}{3}$$

Value is represented as $V$, which defines the strength of the color. $S$ represents the saturation levels of the colors presented in the image. Users’ linguistically based queries can be answered using the Color moment. The color histogram is a common color feature employed in numerous picture retrieval systems. The color histogram is resistant to rotation and slow changes in the angle of vision, occlusion, perspective axis, and size.

#### FIE-Texture

Another aspect of a picture that is used in computer vision and pattern recognition is texture. A recurring pattern of one or more parts in various relative spatial places creates texture, described as the surface structure. The repetition includes regional changes in the elements’ scale, position, or other geometrical and visual characteristics. The capacity to match texture similarity is frequently helpful in identifying between portions of photos with similar hues. Alternative techniques for retrieving textures include using Haar wave-lets transforms. The comparative brightness of particular pixel pairs from each image. Calculations can be made to determine their degree of difference, hardness, positional precision, consistency, regularity, directional cues, and unpredictableness.

##### Haar-based wave-lets transformation

Wavelet transforms offer a multiresolution method for classifying and analyzing textures. A function is represented by the wavelet transform as a combination of a wavelet family of fundamental operations. A two-dimensional image’s wavelet change calculation also uses recursive filtering and subsampling as part of its multiresolution method. The idea is divided into four frequency sub-bands at each level: LL, LH, HL, and HH, where $L$ stands for low frequency and $H$ for high frequency.

14 $${a}_{i}=\frac{({X}_{i}+{X}_{i+1})}{2}$$

The first part of the $X$ -element array is used to record the average, and the second half is used to store the coefficients. The standards serve as the data source for the wavelet calculation’s subsequent phase. $i$ defines the individual element of the resolution presented in the image, and $a$ represents the feature vector of an image queried. The data set’s odd and even elements can be used to produce an average and a wavelet coefficient using the Haar equations.

### Hierarchical layered & similarity indexing system with medical imaging AI systems and big data analytics

From the previously mentioned FIE, the color and texture attributes of the image are calculated. Image delineation is only one factor in a CBIE’s effectiveness; feature indexing and a similarity measurement matrix are also crucial for facilitating query execution. Generally, a feature index refers to an organizational database framework that facilitates quick Retrieval. To address the problems with knowledge discovery on a large-scale dataset, it is still possible to get data from small data sets by comparing the striking similarities between such a seek and each photo in the dataset. The database indexing that has been introduced intends to organize and structure the picture database into a straightforward but efficient type of data groupings and hierarchies. This study’s methodology is substantially different. Based on the mean frequencies of the cluster centers, data groups at higher layers reflect one or more groups at a lower layer in hierarchically layered data clusters. The first layer of image clusters is generated based on feature representations calculated from the Neural Network model. Data cluster groups are formed by combining the related data points using a partition-based clustering approach in CFNN, even if the idea of optimizing Retrieval by constructing hierarchical structures has been considered.

**Fig. 4:**
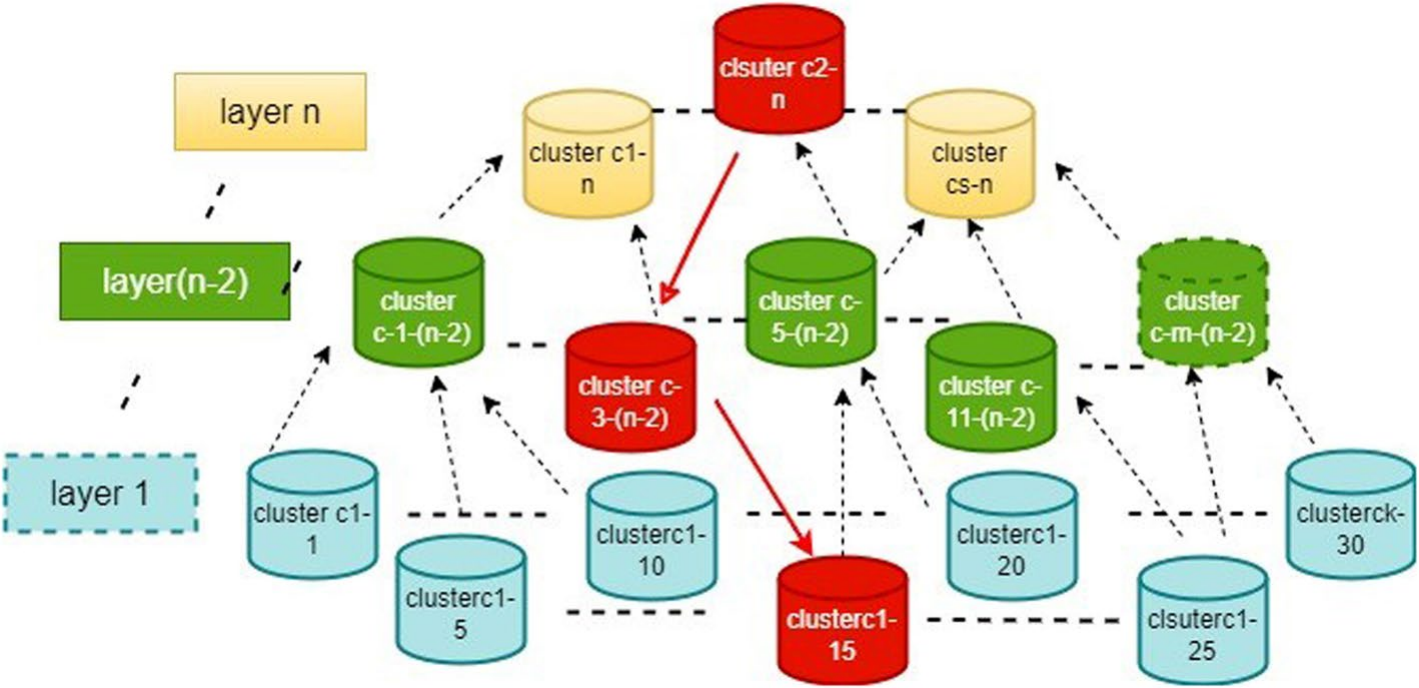
The Hierarchical Layered & Similarity Indexing System with medical imaging AI systems and big data analytics

In Fig. 4 above, the hierarchical and similarity indexing system for image information extraction is organized based on the below-mentioned equations. The relative localized density concerning the query picture, defined by an appropriate kernel range between the present individual frame and all of the other images within the cluster, measures the level of similarity between both the query image and images inside each collection:

15 $$D_c^i=k\left({\textstyle\sum_{j=1}^{M^c}}d_{ij}^C\right)$$

16 $$c=[1,C]$$

Where ${M}^{c}$ Are the associated images count with the cluster ${c}^{n}$, the distance between the image queried and the real picture of the particular ${c}^{th}$ cluster is represented by ${{d}_{ij}}^{C}.$ Total number of images extracted in the ${c}^{th}$ cluster is denoted by N. A hierarchical nesting can utilize a variety of distance measurements, including Euclidean and Cosine distances. By using a kernel of the Cauchy type to specify the local density ${D}_{c}^{i}$. It is demonstrable that the Cauchy kind kernel can be calculated but asymptotically leads to Gaussian.

17 $$D_c^i=\frac1{1+{\Arrowvert F_i-\mu_i^c\Arrowvert}^2+X_i^c-{\Arrowvert\mu_i\Arrowvert}^2}$$

Where $F=\{{f}_{1},{f}_{2},{f}_{3},\dots .{f}_{2048}\}$ is the feature vector. The Mean Value of a similar image is represented by ${\mu }_{i}$. Scalar product is mentioned by the ${X}_{i}$ Need to be updated recursively.

18 $${\mu }_{i}=\frac{i-1}{i}{\mu }_{i-1}+\frac{1}{i}{F}_{i} ,{\mu }_{1}={F}_{1}$$

Concerning the query image, the cluster with the highest local density ${D}^{c}$. It is most likely to include related images.

19 $${C}_{i}^{*}={argmax}_{c=1}^{C}\left\{{D}_{i}^{c}\right\}$$

In Eq. 19 above, ${C}_{i}^{*}$ Represents the overall city block distance of the particular cluster in a group of related images. The query image is compared to every shot in the powerful team at the lowest layer in the last step. The significance score is calculated using the City Block distance for distance-based grading. The scores they earned are then used to decide the order of the photographs. The relevant and query images are more comparable when the City Block distance is shorter and vice versa.

## Experimental results

Since Synthetic Aperture Radar (SAR) imaging systems are not impacted by weather and can thus be utilized both at night and throughout the day., Remote Sensing (RS) of the environment research. It has examined their advantages for various land and maritime applications. It is necessary to test and validate image processing methods on actual and fake images when designing them for synthetic aperture radar applications. The design and development of algorithms to cope with SAR data are supported by benchmark databases from https://ieee-dataport.org/. As a result, it offers and finances experiments for regional, national, and international geoscience and remote sensing contests [25]. The technique is shown to restore synthetic aperture radar with good accuracy. Use domain-specific datasets to train and test neural network models to optimize them for CBIE. The question of whether such networks can be employed as an overall image feature extractor is uncertain. Focusing on new optimization techniques for Synthetic Aperture Radar (SAR) images.

Pritpal Singh et al. proposed a new algorithm based on the mixture of FFQOA and CNN in 2023, which is called FFQoA ConnectTwo. In this algorithm, FFQOA searches for the optimal weights associated with layers by simultaneously achieving the minimum classification error. The application of FFQOAconNetwork in image classification is demonstrated by using benchmark data sets. The empirical analysis shows that compared with other algorithms, FFQOAconNetwork can effectively solve the MOOP problem [26]. The main purpose of FFQOAK proposed by Pritpal Singh et al. in 2021 is to segment the CT scan image of the chest, so that the infected area can be accurately detected. The proposed method is verified by using different chest CT scan images of COVID-19 patients [27, 28].

Simultaneous parameter estimation and model selection are used to illustrate how the model selection process works on textured and untextured areas of the image; the flying image was chosen. In contrast to the previous examples, the attributes of more than one model are no longer dominant in the reconstructed image. Instead, a greater diversity of visual elements can be successfully described and rebuilt using many models. The findings of the studies examine how various factors impact retrieval efficiency.

The report provides a list of assessment methods and is available to the public statistics. To facilitate assessing the RS features extracted quantitatively, the coverage- ratio and Average Mean Precision values obtained after the remote sensing image database underwent 180 trials with the following settings: with the I ranges from $\{\text{1,2},\text{3,4},\text{5,10,20}\}$ samples.

**Fig. 5:**
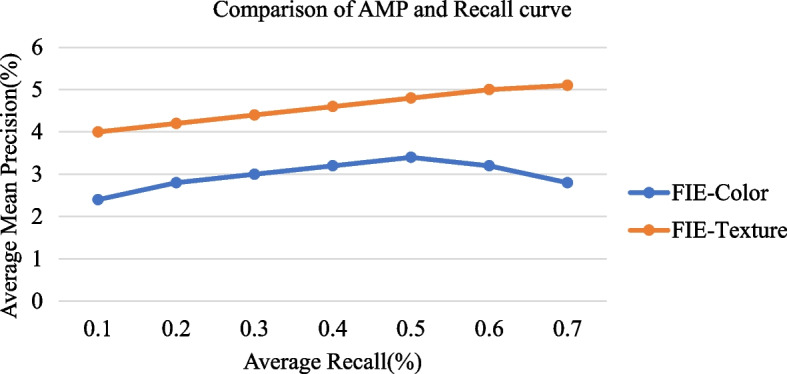
Difference between the AMP and recall graph for CFNN-based FIE

As shown in Fig. 5, with the FIE methods’ help, the image information extraction outcomes using the Color and texture attribute in the Average Mean precision-to-recall ratio are calculated from Eq. 6. Precision is inversely correlated with recall. Precision tells us how well our CFNN model predicts a specific image feature information FIE-color and FIE-texture. The horizontal x-axis recall measure gives quantitative information about our CFNN model’s ability to detect a particular feature, like the color or texture of an image. Modeling of moment aggregate scaled information for several hazy images is obtained from Eq. 7. CFNN-based FIE - Color images feature vector characteristics are analyzed from Eqs. 12 & 13. At the same time, the FIE-Texture information of queried images is calculated from Eq. 14.

**Fig. 6:**
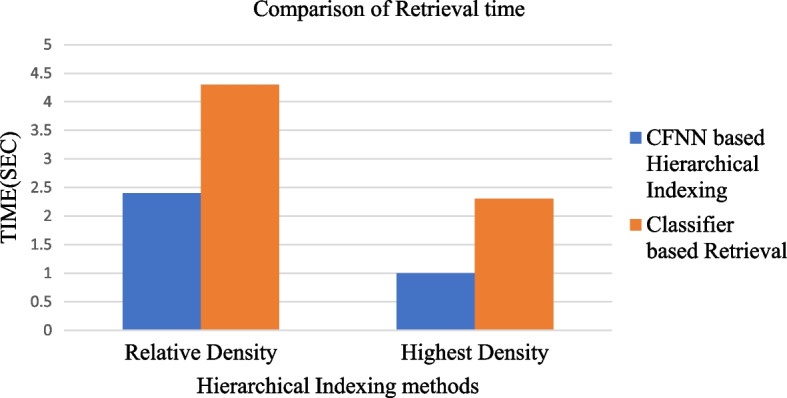
Comparison of retrieval time using Hierarchical Indexing System based on CFNN with CR

Figure 6 shows the fastest retrieval performance attained using the suggested hierarchical indexing strategy based on the CFNN method. Compared to conventional Classifier-based Retrieval of image information extraction, Content-Based Remote Sensing integrated with CFNN achieves a fast retrieval speed of image information from a database.

The method Relative Local Density value of an image is retrieved from Eqs. 15 and 17. Similarly, the Highest Local Density method accesses image information from Eqs. 18 & 19. As a result, computing similarity for every image in the stored database requires additional time. The conventional CR method poses a larger retrieval time than our proposed CFNN integrated with a hierarchical indexing system. The proposed method takes only 1 s to retrieve image information from the queried database.

**Fig. 7:**
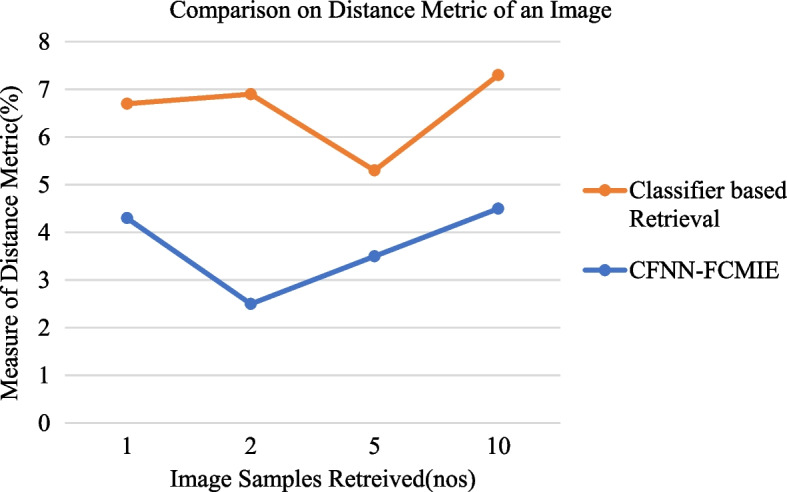
Comparison between CFNN-FCMIE and CR based on distance metric for image information

From the above Fig. 7, the Comparison between CFNN-FCMIE and CR based on Distance Metric for an Image Information Extraction is depicted from the freely available RS image dataset mentioned in [25]. The Distance Metric identified from the number of image samples retrieved from the database is analyzed by Eqs. 4 and 5. CFNN-FCMIE method shows the least distance weight matrix for an image sample collected. At the same time, existing Classifier-based Retrieval consumes a large amount of weighted distance.

**Fig. 8:**
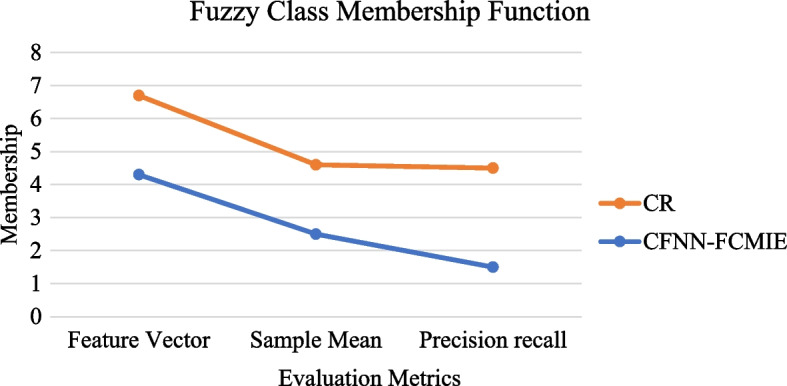
Comparison between the Evaluation metrics of sample image information extraction based on Membership

The below-depicted Fig. 8 represents the Comparison between the Evaluation metrics of sample image information extraction based on the Membership function with various input variables of an image retrieved from [25]. The horizontal values represent the evaluation metrics like the feature vector of an image sample, the sample mean representation, and the precision-recall value of the information retrieved. These values are calculated from Eqs. 1 and 2.

In Fig. 8 above, the conventional CR method poses the highest functional output ranges, approximately 7% of all three metrics, which is unsuitable for efficient image information extraction. The performance of the Compensated Fuzzy Neural Network(CFNN) used to extract visual information is correlated with the graph structure of the membership function. After image information extraction during training, the functional lines are displayed by the membership functions of the input variables a and b. The graphic shows how the input variable’s membership function’s center and width have altered due to image information extraction. The functional membership values are changed for each parameter based on the three different evaluation metrics. The proposed method achieves a minimal value range of 4–5% for all three metrics compared to what has been acquired from Eq. 3.

## Conclusion

Medical imaging AI systems and big data analytics have attracted much attention from researchers of industry and academia. The application of medical imaging AI systems and big data analytics play an important role in the technology of content based remote sensing (CBRS) development. For image information retrieval in CBIE, many color and texture aspects are investigated. Remote sensing can considerably improve retrieval performance regarding coverage ratio and Average Mean Precision. For image retrieval in CBIE, many color and texture aspects are analyzed. The proposed method CFNN-FCMIE achieves a minimal value range of 4–5% for all three feature vectors, the sample means, and the precision-recall rate of Comparison, which gives better results than the existing Classifier based Retrieval model. The strength of the CFNN-FCMIE method improves the retrieval performance of image information effectively, and a hierarchical structure was used in the model’s construction; hence it is scalable. So, a dynamic image dataset can be hierarchically handled via feature indexing, and integration is straightforward. The future enhancement is to retain the computational effectiveness of the querying process while preserving multi-dimensional and highly discriminative image representations produced by the CFNN model integrated with the RS field. The work serves an important reference to medical imaging AI systems and big data analytics. Accuracy is inversely proportional to recall. Accuracy tells us the effect of CFNN model in predicting five colors and five textures of specific image feature information. The retrieval time of CFNN integrated with hierarchical indexing system is shortened to 1 s. The method in this paper achieves a minimum range of 4–5% for all three metrics.

## Data Availability

The figures used to support the findings of this study are included in the article.
